# Bardoxolone-Methyl (CDDO-Me) Impairs Tumor Growth and Induces Radiosensitization of Oral Squamous Cell Carcinoma Cells

**DOI:** 10.3389/fphar.2020.607580

**Published:** 2021-01-29

**Authors:** Cornelius Hermann, Simon Lang, Tanja Popp, Susanne Hafner, Dirk Steinritz, Alexis Rump, Matthias Port, Stefan Eder

**Affiliations:** ^1^ Bundeswehr Institute of Radiobiology, Munich, Germany; ^2^ Bundeswehr Institute of Pharmacology and Toxicology, Munich, Germany; ^3^ Institute of Pharmacology of Natural Products and Clinical Pharmacology, University of Ulm, Ulm, Germany; ^4^ Institute and Outpatient Clinic for Occupational, Social and Environmental Medicine, Inner City Clinic, University Hospital of Munich (LMU), Munich, Germany

**Keywords:** oral squamous cell carcinoma, radiotherapy, reactive oxygen species, heme oxygenase-1, bardoxolone-methyl

## Abstract

Radiotherapy represents a common treatment strategy for patients suffering from oral squamous cell carcinoma (OSCC). However, application of radiotherapy is immanently limited by radio-sensitivity of normal tissue surrounding the tumor sites. In this study, we used normal human epithelial keratinocytes (NHEK) and OSCC cells (Cal-27) as models to investigate radio-modulating and anti-tumor effects of the synthetic triterpenoid 2-cyano-3,12-dioxooleana-1,9,-dien-28-oic acid methyl ester (CDDO-Me). Nanomolar CDDO-Me significantly reduced OSCC tumor xenograft-growth *in-ovo* applying the chick chorioallantoic membrane (CAM) assay. In the presence of CDDO-Me reactive oxygen species (ROS) were found to be reduced in NHEK when applying radiation doses of 8 Gy, whereas ROS levels in OSCC cells rose significantly even without radiation. In parallel, CDDO-Me was shown to enhance metabolic activity in malignant cells only as indicated by significant accumulation of reducing equivalents NADPH/NADH. Furthermore, antioxidative heme oxygenase-1 (HO-1) levels were only enhanced in NHEK and not in the OSCC cell line, as shown by immunoblotting. Clonogenic survival was left unchanged by CDDO-Me treatment in NHEK but revealed to be abolished almost completely in OSCC cells. Our results indicate anti-cancer and radio-sensitizing effects of CDDO-Me treatment in OSCC cells, whereas nanomolar CDDO-Me failed to provoke clear detrimental consequences in non-malignant keratinocytes. We conclude, that the observed differential aftermath of CDDO-Me treatment in malignant OSCC and non-malignant skin cells may be utilized to broaden the therapeutic range of clinical radiotherapy.

## Introduction

Malignancies of the oral cavity are among the most common cancers within the European Union. According to estimates of the European Cancer Information System (ECIS) over 45,000 cancer cases of the lip and oral cavity were diagnosed in 2018, representing a crude incidence rate of 8.9 per 100,000 (Likhtarev et al., 2006). Despite advances in modern multidisciplinary treatment modalities comprising surgery, radio-chemotherapy and targeted pharmacological therapy, the overall outcome of oral squamous cell carcinoma (OSCC) patients still remains dissatisfying (De Felice et al., 2018). Therefore, scientific efforts have previously been made to overcome clinical limitations and side-effects of OSCC treatment regimes. The therapeutic window for radiotherapy is mainly narrowed by local side effects mainly due to damage of surrounding normal tissue when targeting cancer sites. Aside from the recent implementation and constant advancement of intensity-modulated radiotherapy (IMRT), a further strategy to restrict radiation doses for neighboring normal cells lies within the identification of small-molecule drugs allowing for the radio-sensitization of cancer cells and ideally with a radio-protective effect on healthy tissue (Lindemann et al., 2018; Ho et al., 2019; Morra et al., 2019).

The synthetic oleanane triterpenoid 2-cyano-3,12-dioxooleana-1,9,-dien-28-oic acid (CDDO) and its C-28 methyl ester (CDDO-Me, Bardoxolone-methyl) has been shown to exert beneficial therapeutic activities by suppressing inflammation and oxidative stress *in vitro* and *in vivo* at low nanomolar concentrations (Liby and Sporn, 2012). The BEACON-study (ClinicalTrials.gov Identifier: NCT01351675), a randomized, placebo-controlled phase 3 clinical trial, evaluated CDDO-Me induced effects on the kidney function in 2,185 patients suffering chronic kidney disease and type 2 diabetes. Although the study ultimately had to be terminated due to increased rates of heart failure events, CDDO-Me revealed to increase eGFR and to significantly reduce the hazard for the loss of kidney function (Chin et al., 2018). Besides the inhibition of the nuclear factor κB (NFκB) signaling cascade, activation of the Kelch-like ECH-associated protein 1 (Keap1)/nuclear factor erythroid 2–related factor (Nrf2) pathway is widely regarded as a major mechanism of action for CDDO-Me related cytoprotective effects (Liby and Sporn, 2012; Wang et al., 2014). Stimulation of the Nrf2 pathway mediates the downstream activation of various promoter genes encoding for detoxifying and antioxidative proteins like heme oxygenase 1 (HO-1). The heat-shock protein (HSP)-32 family member HO-1, which has been found in microsomes, mitochondria and nuclei, was demonstrated to catalyze the rate-limiting step of heme catabolism, leading to the formation of biliverdin. The following biliverdin/bilirubin redox cycle system effectively scavenges reactive oxygen species (ROS) and represents a highly conserved cellular control mechanism against oxidative stressors like radiation (Lin et al., 2007; Kim and Park, 2012; Son et al., 2013).

Numerous experimental studies highlighted the efficacy of CDDO-Me for both, prevention and treatment of cancer, albeit predominantly at high nanomolar to micromolar concentrations (Liby and Sporn, 2012; Borella et al., 2019).

However, differential reactions to radiation exposure of normal and cancer cells at equivalent and physiological achievable CDDO-Me concentrations are preferably required when giving consideration to a future usage in radiotherapy. Previously, CDDO-Me has been demonstrated to mitigate radiation-induced damage in normal epithelial cells but not cancer cells of the lung, breast and colon (Kim et al., 2013; El-Ashmawy et al., 2014).

In this study, we analyzed the implications of low nanomolar CDDO-Me in the radiation response and *in ovo* tumor growth of the OSCC cell line Cal-27 and compared the results with findings in normal human epithelial keratinocytes (NHEK) as a model for surrounding healthy skin.

## Materials and Methods

### Cell Culture and Treatment

Cal-27 cells were originally derived from a 56-year old male patient suffering SCC of the tongue and were purchased from Leibniz-Institut DSMZ (Braunschweig, Germany). Cells were cultivated at 37 °C in a 5% CO_2_ atmosphere using DMEM GlutaMAX medium (Gibco, Eggenstein, Germany), which was supplemented with 10% FCS (Boehringer, Mannheim, Germany).

Primary normal human epidermal keratinocytes (NHEK) originate from the epidermal stratum basale of an adult single donor and were cultivated at 37 °C and 5% CO_2_ in Keratinocyte Growth Medium 2 (both from PromoCell, Heidelberg, Germany).

Unless stated differently, seeded cells were allowed to attach for 24 h, then culture medium was supplemented with 10 nM CDDO-Me or DMSO as solvent control at 0.1 vol% (both from Selleckchem, Houston, United States) and cells were incubated for further 6 h. Subsequently, cells were treated according to the respective protocol.

### Radiation Exposure

Cells were exposed to 240 kV X-rays using the YXLON Maxishot (Hamburg, Germany) including a 3 mm beryllium filter at a plateau dose rate of 1 Gy/min at 13 mA. Monitoring of the applied doses was performed by a PTW Unidose dosimeter (PTW Freiburg GmbH, Freiburg, Germany).

### Chick Egg Chorioallantoic Membrane as Tumor Xenograft Model

The chick egg chorioallantoic membrane (CAM) tumor model was used as previously described (Zuo et al., 2017; Kuan et al., 2018; Hafner et al., 2019). Briefly, fertilized chicken eggs were incubated at 37 °C and 60% relative air moisture for 7 days before fenestration and placement of a silicone ring (diameter 5 mm) on the vascularized CAM. Cal-27 cells were treated with 10 nM CDDO-Me or DMSO respectively 6 h prior to IR exposure and subsequently harvested. A 1:1 solution of matrigel (BD, Heidelberg, Germany) and medium containing Cal-27 cells (1.5 × 10^6^ cells/egg) was grafted within the ring. The following day, topical treatment with CDDO-Me (10 nM) or vehicle (0.2% DMSO in NaCl 0.9%) was started and continued for two more days. After an incubation period of 4 days at 37 °C, tumors were collected, imaged, fixed in phosphate-buffered 4% formaldehyde solution and embedded in paraffin for immunohistochemical analysis. Slices (5 µm) were stained for H and E and proliferation marker Ki-67 (Dako, Glostrup, Denmark). Mean tumor volume of Cal-27 cells cancer xenografts was assessed immediately after extraction. Tumor volume was calculated according to the formula: π/6 x length x width^2^ (Tomayko and Reynolds, 1989).

### Assessment of Cellular Reactive Oxygen Species

In order to show whether CDDO-Me decreases the amount of free reactive oxygen species (ROS) after irradiation within NHEK and Cal-27 cells, we used the DCFDA Cellular ROS Detection Assay Kit according to the manufacturer’s instructions (Abcam, Cambridge, United Kingdom). In brief, 2′, 7’–dichlorodihydrofluorescein diacetate (DCFDA) served as a marker which accumulates in living cells and becomes fluorescent upon oxidation. Therefore, 0.5 × 10^6^ cells (Cal-27) or 0.75 × 10^6^ cells (NHEK) were incubated for 24 h in 6 cm diameter Petri dishes. Subsequently, 1 μL/ml medium CDDO-Me stock solution (10 µM in DMSO) was added to the treatment group resulting in 10 nM CDDO-Me, whereas 1 μL/ml medium DMSO was added to the control group and incubated for another 6 h before undergoing X-ray irradiation; 45 min before irradiation, cells were stained with 25 µM DCFDA; 55 mM Tert-Butyl Hydrogen. Peroxide (TBHP) served as positive control. Immediately after irradiation the cells were detached by trypsinization and measured by flow cytometry using the FACS-Calibur System (BD Biosciences, Franklin Lakes, NJ, United States). Therefore, 10,000 objects per sample were recorded on FL-1 (535 nm) with an excitation wavelength of 488 nm. Single cells were gated via a “forward scatter vs side scatter” scatterplot and the mean intensity of FL-1 was taken as measured value. Experiments were performed in quadruplicate (10,000 cells/experiment). Furthermore, cells were grown on chamber slides for live cell fluorescence imaging. According to the manufacturer’s protocol (Abcam) cells were washed, stained with DCFDA and the developing fluorescence was captured by a Zeiss Axioimager 2i fluorescence microscope.

### Redox Status

For analysis of the cellular redox homeostasis we used the CellTiter 96® AQueous One Solution Cell Proliferation Assay according to the manufacturer’s instructions (Promega, Madison, USA). The assay is based on the bioreduction of MTS [3-(4,5-dimethylthiazol-2-yl)-5-(3-carboxymethoxyphenyl)-2-(4-sulfophenyl)-2H-tetrazolium] to a colored formazan by NADPH or NADH produced in metabolic active cells. Absorbance at 490 nm indicated the amount of formazan formed using the Multiskan™ FC microplate photometer (Thermo Scientific, Westham, United States).

### Immunofluorescence Microscopy

We applied immunocytochemistry as described previously (Liebau et al., 2011). Rabbit monoclonal anti-HO-1 (dilution 1:1,000, Cell Signaling, Danvers, United States) served as primary antibody before fluorescence-labeling using Alexa Fluor® 488-conjugate goat polyclonal anti-rabbit (dilution 1:500, Life Technologies, Waltham, United States). Cytoskeleton was stained using TexasRed-conjugated Phalloidin (dilution 1:40, Invitrogen, Mannheim, Germany) and nuclei were counter stained using Fluoroshield Mounting Medium (Abcam, Cambridge, United Kingdom) containing 4,6-diamidino-2-phenylindole (DAPI).

For image acquisition we used a Zeiss Axioimager 2i fluorescence microscope in combination with the ISIS fluorescence imaging system (MetaSystems, Altlussheim, Germany).

### Immunoblotting

The XCell Sure Lock™ Mini-Cell Electrophoresis System served as a platform for western blot experiments according to standard protocols. For equalization of protein concentrations, we used the BCA Protein Assay Kit (both from Thermo Scientific, Westham, United States). Amounts of HO-1 were detected using primary rabbit monoclonal anti-HO-1 (dilution 1:1,000, Cell Signaling, Danvers, United States) and secondary HRP-conjugated polyclonal goat anti-rabbit (dilution 1:10,000, Thermo Scientific, Westham, United States). For digital image acquisition we used the myECL™ Imager system (Thermo Scientific, Westham, United States). For calculation of HO-1/GAPDH-ratios greyscale intensity values were determined by ImageJ software, v. 1.51 (NIH, Bethesda, United States).

### DNA Double Strand Break Analysis Using Imaging Flow Cytometry

DNA double strand breaks were assessed using phosphohistone γH2AX as a marker. Cells were detached, fixed for 20 min in cold 4% PFA pH 7.0 (Roti-Histofix®, Carl Roth, Karlsruhe, Germany), washed twice, permeabilized for 10 min using 0.1% Triton X (Sigma Aldrich, Darmstadt, Germany) and washed twice again**.** Staining was performed for 2 h at room temperature using mouse γH2AX antibodies primarily coupled with AlexaFluor® 488 (BioLegend, San Diego, CF, United States). After one additional washing step, cells were resuspended in 100 µl PBS, containing 20 µM DRAQ5 for DNA staining, yielding at least 10^6^ cells/ml and measured using the ImageStream® X mkII (Luminex, Austin, TX, United States) imaging flow cytometer (IFC).

Excitation lasers with 488 nm and 642 nm wavelength were used at laser powers adjusted to the sample with the highest expected signal for each data set. The emission wavelengths recorded, were split into different channels on the CCD camera of the IFC. Channel 1 (435–480 nm) was used for the bright field picture and therefore illuminated with a LED of the respective wavelength range; Channel 2 (480–560 nm) recorded the green fluorescent AF488 emission and Channel 5 (642–745 nm) recorded the DRAQ5 signal. The remaining channels were not used; notch filters were activated to block laser scatter from the CCD.

Analysis of γH2AX foci was performed by the spot count feature on custom masks (Range (Peak (M02, Ch02, Bright, 10), 4–100, 0–1))**.**

### Clonogenic Survival Assay

NHEK and Cal-27 cells were cultivated in 6-well plates for 24 h and treated according to the standard protocol. Experiments were stopped after 9 days by fixing cells with 70% ethanol followed by staining with gentian violet. We counted colonies manually by using a Zeiss STEMI SV8 stereomicroscope. Experiments were performed in quadruplicate.

### Analysis of Cell Growth and Proliferation

Proliferation was determined by the IncuCyte™ live-cell imaging system (IncuCyte S3, Essen BioScience, United States). Cells (50,000 NHEK cells/12 well; 25,000 Cal-27 cells/12 well) were pre-incubated with CDDO-Me (10 nM) or the solvent control DMSO for 6 h before irradiation. Every 2 h a picture was taken to monitor cell growth for 48 h. The integrated IncuCyte software was used to measure the increase of confluence which was normalized to the initial confluence values.

### Statistics

Unless stated elsewhere we tested for significance using one way ANOVA followed by post hoc Bonferroni *t*-test using SigmaPlot software (v. 14.0, Systat Software, Erkrath, Germany). Regarding clonogenic survival assays we calculated plating efficiency (PE) and surviving fraction (SF) as followed: PE = (Colonies counted)/(Cells seeded per well)*100; SF = (Colonies counted)/((cells seeded per well) (PE/100)). The reference basic value (100%) represents the mean SF of untreated control group (DMSO, 0 Gy).

*p*-values < 0.05 were regarded as statistically significant. Bars indicate mean values ±standard deviation, unless stated elswhere.

## Results

### CDDO-Me Impaired Tumor Forming Capability of OSCC Cells

**FIGURE 1:**
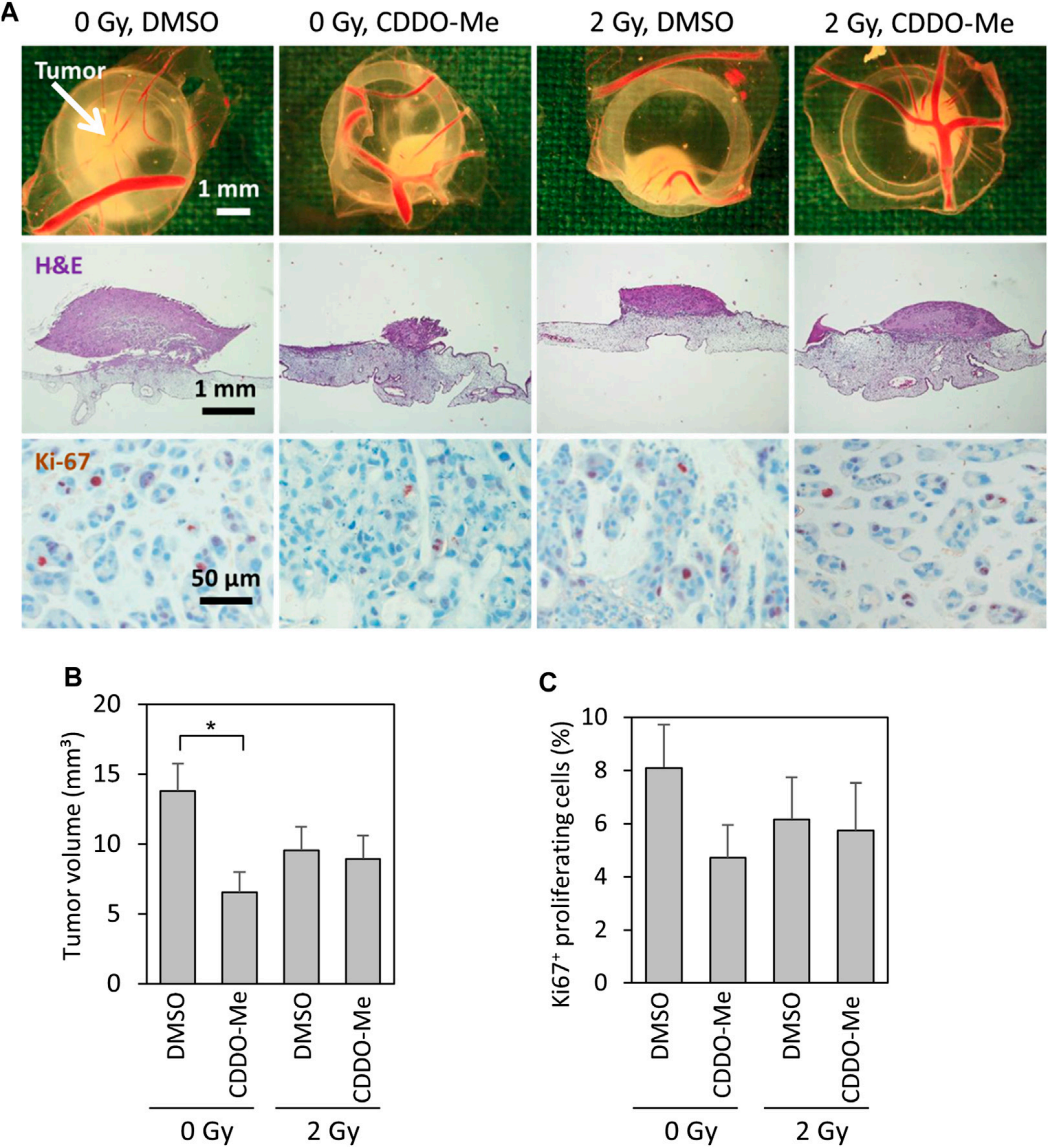
10 nM CDDO-Me inhibits growth of Cal-27 cell xenografts on the chick egg chorioallantoic membrane *in vivo* **(A)** Representative pictures of tumor xenografts immediately after extraction (first row), overview of tumor and underlying CAM tissue (H and E stained, second row), immunohistochemical staining of Ki-67 + proliferative cells (third row) **(B)** Mean tumor volume of Cal-27 cell cancer xenografts as assessed immediately after extraction. Data are mean ± SEM of 13–15 tumors/group. Statistics: One-way ANOVA, post hoc test: Bonferroni *t*-test; *p < 0.05 **(C)** Percentage of proliferating Ki-67 + cells. 226–1,072 cells of each tumor were evaluated. Data are mean ± SEM of 9–13 tumors/group.

Cal-27 cells treated with CDDO-Me 6 h before receiving 2 Gy ionizing radiation (IR) were subsequently implanted on vascularized chick egg chorioallantoic membranes to test the inhibitory capacity of the triterpenoid on tumor growth. After 4 days the volume of CDDO-Me treated tumors was significantly reduced compared to untreated controls (Figures 1A,B). The fraction of Ki67-proliferative cells within the tumor tissue revealed to be reduced by trend even though without reaching statistical significance levels (Figures 1A,C). Surprisingly, combined treatment of IR and CDDO-Me did not show any additive effect (Figures 1B,C).

### Treatment With CDDO-Me Affects ROS Levels and Antioxidative Response

The unexpected absence of synergistic effects in the *in ovo* model prompted us to focus on the role of CDDO-Me itself first. Analysis of CDDO-Me cytotoxicity exhibited IC_50_ values of 820 nM in NHEK and 280 nM in Cal-27 cells (data not shown). In order to use CDDO-Me in its low nanomolar effective range in all experiments 10 nM of the substance were used.

**FIGURE 2:**
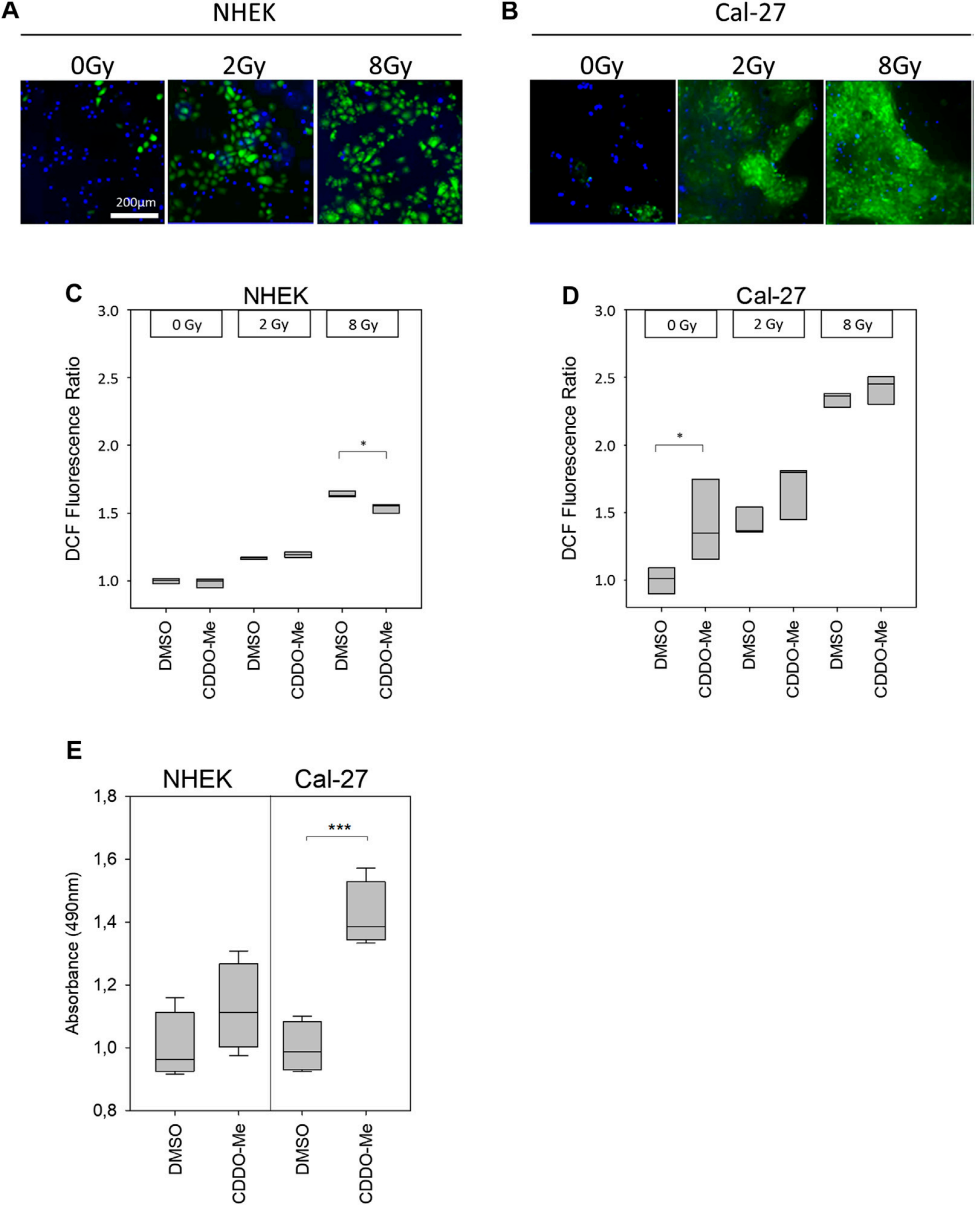
**(A and B)** Cellular ROS-sensitive DCF levels (green) of NHEK and Cal-27 cells increased in a dose-dependent manner as shown by representative fluorescence microscopy images. Nuclear DAPI staining (blue) was used to indicate the presence of cells **(C and D)** Treatment with 10 nM CDDO-Me was shown to reduce DCF fluorescence intensity within NHEK significantly at high doses (8 Gy). In contrast, solely baseline DCF fluorescence levels of Cal-27 cells appeared to be increased significantly in the presence of 10 nM CDDO-Me. Statistics: Two Way ANOVA, post hoc test: Holm-Sidak method, *p < 0.05; ***p < 0.001 (n = 3) **(E)** Generation of reducing equivalents NADPH or NADH after incubating cells with 10 nM CDDO-Me for 1 h was determined by quantification of MTS formazan product (absorbance at 490 nm). Significance levels were referenced to absorbance at 0 nM CDDO-Me (DMSO). Error bars represent standard deviation. Statistics: One Way ANOVA, post hoc test: Bonferroni *t*-test, *p < 0.05; ***p < 0.001 (n = 4).

**FIGURE 3:**
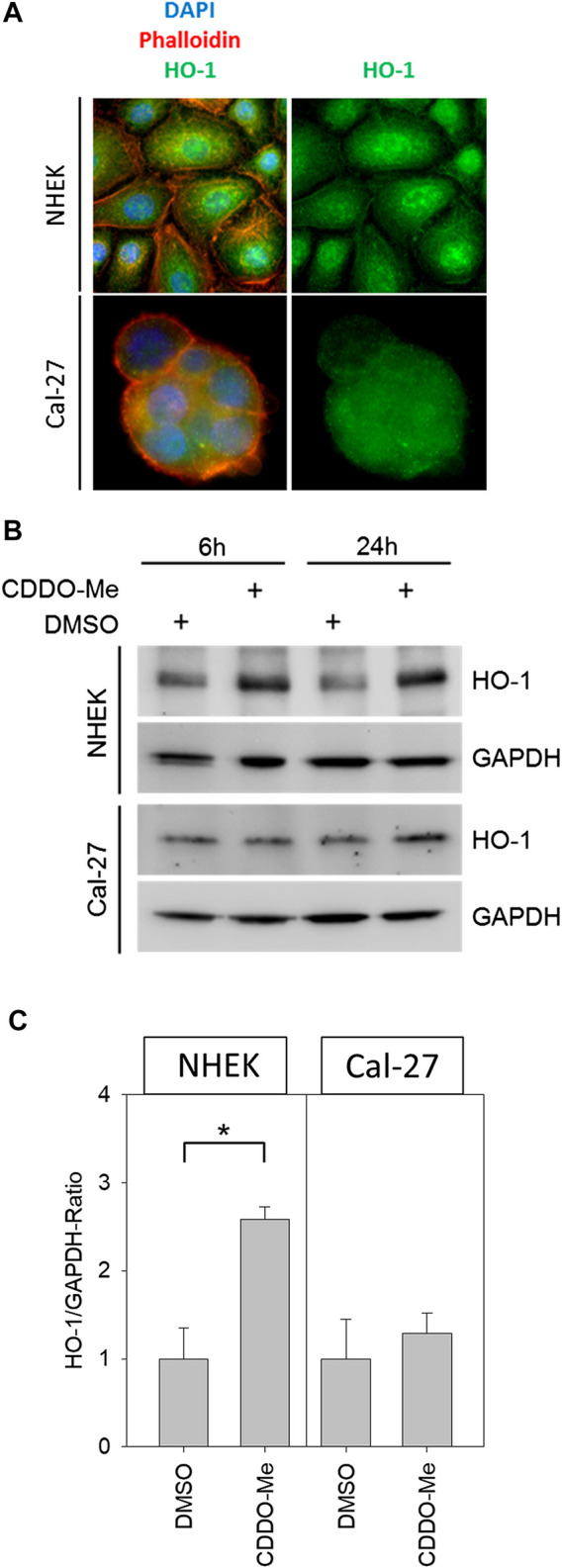
**(A)** Representative immunofluorescence pictures of NHEK and Cal-27 cells; stained structures are the nucleus (DAPI, blue), actin (Phalloidin-dye conjugate, red) and HO-1 (FITC-Antibodies, green) showing that HO-1 is present ubiquitously in both cell lines **(B)** Western blots of HO-1 and GAPDH from control and treated NHEK and Cal-27 cells **(C)** HO-1/GAPDH ratio determined by western blot; compared groups are control (DMSO) vs. 10 nM CDDO-Me treatment (incubation 6 h and 24 h). HO-1 in NHEK treatment group is significantly increased vs. control, whereas the increase in Cal-27 cells is not significant. Statistics: Welch's *t*-test *p < 0.05; n = 3.

Cellular reactive oxygen species (ROS) levels were detected by monitoring the oxidation of the fluorescent probe DCFDA. Cells, which were stained with the probe, irradiated and subsequently examined under the microscope, revealed a dose-dependent increase of fluorescent signal in microscopic analysis (Figures 2A,B). Further investigation of ROS activity was based on flow cytometric measurement. While baseline ROS activity was found to be unaltered in the presence of CDDO-Me in non-malignant NHEK (Figure 2C), Cal-27 cells responded with significantly increased ROS production during CDDO-Me treatment (Figure 2D) which is comparable to the effect of 2 Gy IR. However, no verifiable additional CDDO-Me induced increase of cellular ROS was detectable when Cal-27 cells were irradiated with 2 Gy or 8 Gy respectively. Similarly, a strong generation of NADPH, a potent provider of reducing equivalents, was observed in Cal-27 cells (Figure 2E). Additionally, since HO-1 is known for its protective effect against oxidative stress we investigated the expression levels in both cell types. Immunofluorescent staining revealed a ubiquitous production of HO-1 in Cal-27 cells and NHEK (Figure 3A). When incubating cells with 10 nM CDDO-Me over 6 h the subcellular localization of HO-1 was left unchanged (data not shown). However, HO-1 levels increased significantly in the whole cell lysate of NHEK in the presence of CDDO-Me as shown by immunoblotting analyzed after 6 and 24 h. In contrast, for Cal-27 cells no significant enhancement of expression level was detected (Figure 3B) as further illustrated by the given ratios of HO-1 and housekeeping protein GAPDH in (Figure 3C).

### CDDO-Me Augmented the Effect of Irradiation in OSCC Cells

**FIGURE 4:**
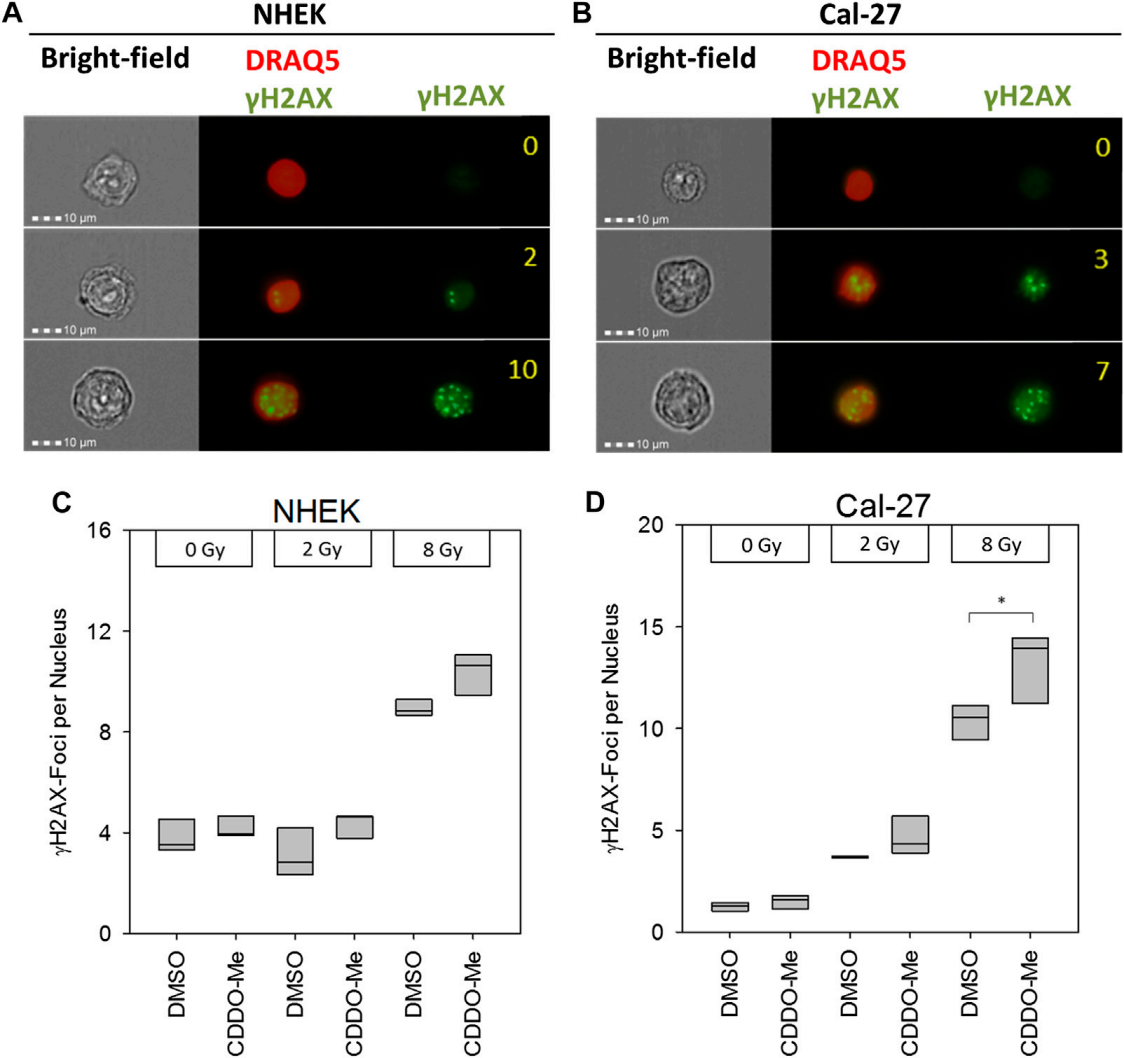
**(A and B)** Representative pictures of NHEK and Cal-27 cells showing different DNA damage levels as indicated by γH2AX staining using the AMNIS™ image stream flow cytometer. Numbers indicate digitally counted γH2AX-foci frequency **(C and D)** 10 nM CDDO-Me induced significantly elevated γH2AX foci 6 h after irradiation in Cal-27 cells only (8 Gy). Statistics: One Way ANOVA, post hoc test: Bonferroni *t*-test, *p < 0.05 (n = 3).

Since CDDO-Me is not only described as anti-cancer drug but also as radioprotector of non-cancerous lung cells (El-Ashmawy et al., 2014) we also evaluated the radiomodulatory capacity of CDDO-Me in our epithelial cell model after irradiation. DNA double strand breaks are among the most detrimental effects of irradiation. Therefore, the number of γH2AX foci was counted by an imaging flow cytometer (Figures 4A,B). Frequency of double strand breaks increased in a dose-dependent manner in Cal-27 cells, whereas in NHEK increased FOCI only occurred with higher doses (Figures 4C,D). Interestingly, CDDO-Me treatment significantly enhanced γH2AX foci frequency following 8 Gy IR in Cal-27 cells only (Figure 4D).

In line with these results, irradiation induced ROS dose-dependently in both cell types (Figures 2C,D). However, in Cal-27 cells CDDO-Me augmented irradiation-induced ROS production only by trend (Figure 2D) whereas in NHEK, irradiated with high doses, CDDO-Me attenuated oxidative stress significantly (Figure 2C).

**FIGURE 5:**
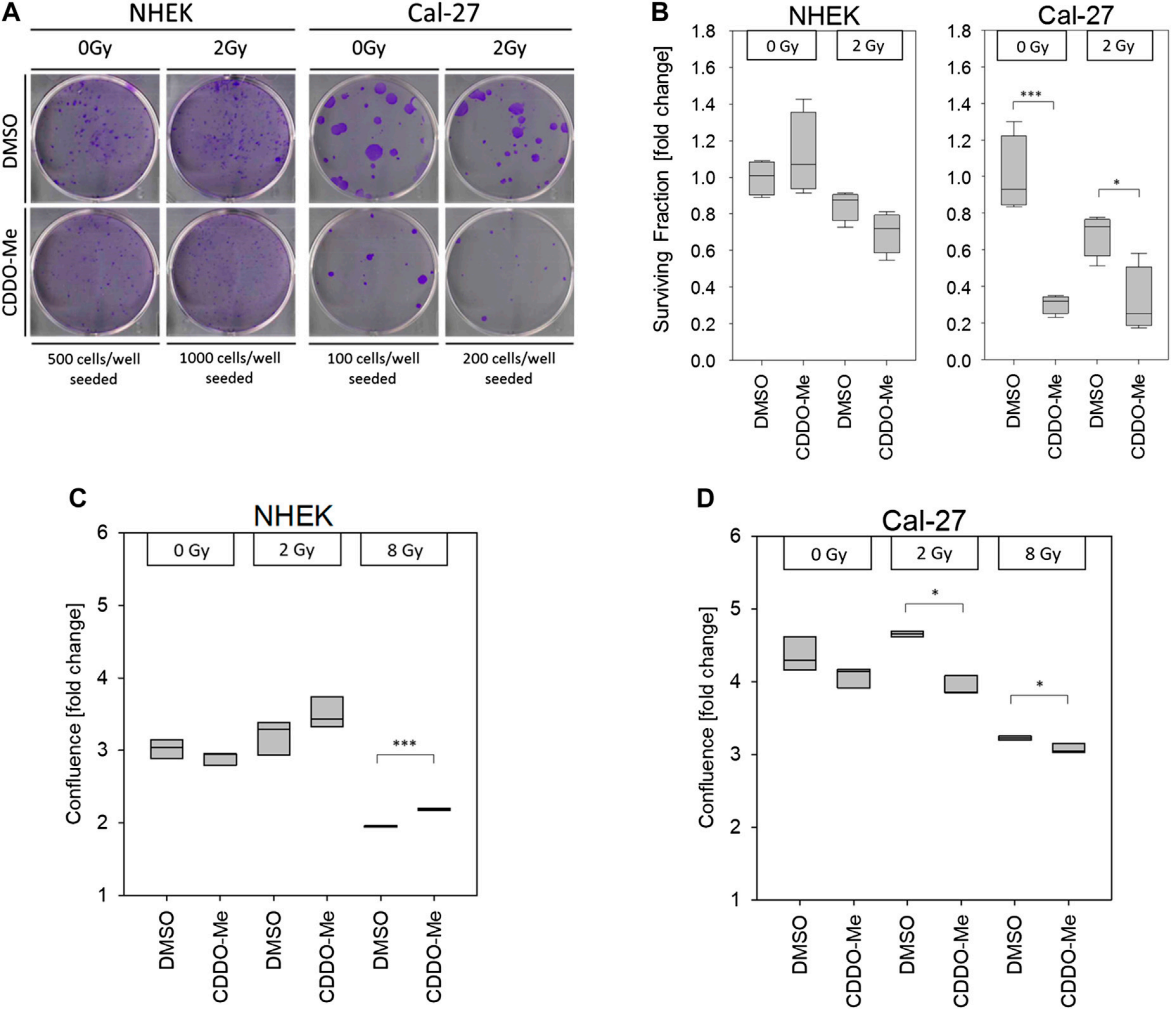
**(A)** Pictures show representative clonogenic survival assays. All experiments were performed in quadruplicate **(B)** 10 nM CDDO-Me induced no significant changes of cellular survival in NHEK, but significantly impaired Cal-27 cell clonogenicity. Statistics: Two Way ANOVA, post hoc test: Bonferroni *t*-test, *p < 0.05; ***p < 0.001 (n = 3) **(C and D)** Cellular confluency levels after a incubation period of 48 h were analyzed by IncuCyte™ live cell imaging experiments. Treatment with 10 nM CDDO-Me 1 h before irradiation showed significantly increased confluency of NHEK at high doses (8 Gy), whereas decreased confluency of malignant Cal-27 cells indicated sensitization to ionizing radiation (2 Gy and 8 Gy). Confluence is displayed as fold change from start of experiment (t = 0 h). Statistics: Welch's *t*-test, *p < 0.05; ***p < 0.001 (n = 3).

As proposed by the CAM assay CDDO-Me has inhibitory effects on OSCC cell proliferation. To assess the survival and the growth capacity of the cells after CDDO-Me treatment clonogenic assays were performed. Cells were treated with CDDO-Me and further cultivated for 9 days. We observed a significant decline in the number of stained Cal-27 colonies (Figure 5A). In accordance, CDDO-Me reduced the surviving fraction in Cal-27 cells by about 70% whereas NHEK surviving fraction remained unaffected (Figure 5B).

Finally, the results from our proliferation studies are in line with the above mentioned data. Both cell types were cultivated for 48 h and confluence was monitored over time in a live cell imaging system. Proliferation of NHEK following 8 Gy IR was significantly increased in the presence of CDDO-Me (Figure 5C) whereas proliferation of irradiated Cal-27 cells was even further impaired if combined with CDDO-Me treatment (Figure 5D).

## Discussion

Over the last decades radiotherapy has traditionally played an essential role in the clinical management of oral squamous cell carcinoma (OSCC). Despite the development of various modifications of conventional radiotherapy regimens, there is still a narrow ridge between effective cancer treatment resulting in improved patient’s outcome and the prevention of adverse effects by damaging neighboring healthy tissue (Glenny et al., 2010). In this study, the triterpenoid CDDO-Me, when used in nanomolar concentrations, significantly impaired tumor forming capability of OSCC xenografts grown on the CAM of fertilized chicken eggs. CDDO-Me treatment surprisingly did not offer a synergistic or additive effect if combined with IR. A possible reason for the lack of the above mentioned effect might be a cellular preselection of Cal-27 cells upon IR exposure prior to implantation on the chicken CAM. Therefore we wanted to investigate the effect of CDDO-Me in detail on OSCC cells, represented by the *in vitro* Cal-27 cell line. To eludicate the effect on healthy neighboring tissue, we used a non malignant primary keratinocyte cell line (NHEK) alongside with the OSCC cells in our *in vitro* experiments.

We demonstrated that CDDO-Me exerts anti-cancer activity in OSCC cells, while not consistently impairing cell homeostasis of primary keratinocytes even in the presence of ionizing radiation. Our findings are in line with previous studies, which highlighted radio-protective effects of CDDO-Me in normal epithelial cells of the lung, breast and colon but not in cancer cells (Kim et al., 2012; Kim et al., 2013; El-Ashmawy et al., 2014).

Activation of the Nrf2 pathway followed by downstream up-regulation of antioxidant enzymes like HO-1 is widely regarded as a major mechanism of action of CDDO-Me (Liby and Sporn, 2012). In physiological settings the Nrf2/HO-1 cascade represents a key mechanism for normal cells to adapt to oxidative stress conditions, mediating enhanced survival, preserved cellular homeostasis and prevention of carcinogenesis (Nitti et al., 2017). Several studies using rodent models and a case report on a HO-1 deficient patient revealed the radio-protective activity of antioxidant HO-1 in normal tissue, including skin (Yachie et al., 1999; Kapturczak et al., 2004; Zhang et al., 2012).

In our study we found that Cal-27 OSCC cells showed significantly increased ROS activity subsequent to CDDO-Me treatment and since NADPH and NADH proved to be enhanced concomitantly upon CDDO-Me administration we hypothesize that CDDO-Me may selectively trigger ROS accumulation in cancer cells via activation of the mitochondrial metabolism as the major source of intracellular ROS. Additionally, NAPDH serves as the donor of reductive potential to glutathione and therefore finally to restore redox homeostasis (Fernandez-Marcos and Nobrega-Pereira, 2016).

No evidence was found for down-regulated antioxidative defensive mechanisms by CDDO-Me as a potential contributing factor for elevated intracellular ROS levels, since HO-1 concentrations revealed to be even increased by trend in OSCC cells. Contrary to the significantly increased HO-1 in NHEK, this observation failed to reach statistical significance, which may in part be referred to elevated baseline HO-1 expression levels in Cal-27 cells. Constitutive up-regulation of antioxidative adaptive mechanisms is widely regarded to be inherent in malignant cells and was shown to be associated with cancer progression and resistance to therapy (Nogueira and Hay, 2013; Nitti et al., 2017).

Our non malignant primary cell line (NHEK) did not show increased ROS activity due to the treatment with CDDO-Me. On the contrary, when exposed to IR doses as high as 8 Gy, there was even a significant reduction in ROS. This data is supported by significantly enhanced HO-1 levels when treating NHEK with CDDO-Me, indicating radioprotective effects of CDDO-Me possibly mediated via enhanced expression of cellular HO-1. Sole CDDO-Me administration left ROS activity in NHEK unchanged, which argues for direct pharmacological effects finally leading to augmented HO-1 instead of elevated HO-1 levels due to increased oxidative stress.

Surprisingly, the diminished ROS levels found after high dose (8 Gy) irradiation were not potent enough to result in measurable reduction of γH2AX-foci of NHEK whereas it was significantly increased in Cal-27 cells. The missing verification of reduced DNA damage subsequent to reduced ROS in NHEK may in part be explained by the saturation of γH2AX-foci induction commonly observed by administration of high radiation doses (Rothkamm et al., 2015). Furthermore, even radiation with low linear energy transfer, such as X-rays generates DNA double strand breaks to some extent via ROS-independent direct ionization of target DNA molecules, representing a way of action that is not preventable by elevated antioxidative cellular defense mechanisms.

On the other hand, significantly reduced ROS activity in NHEK after 8 Gy points to cytoprotective effects by prevention of oxidative damage to various molecular structures crucial for the maintenance of cell homeostasis, e.g., within cellular membranes or organelles.

By all means, low nanomolar CDDO-Me did neither provoke acute toxic effects (IC_50_ = 820 nM) nor impairment of viability, clonogenicity or radioresistance in NHEK. A recent *in vivo* study even highlighted radioprotective effects for healthy skin when radiation-induced dermatitis was shown to be mitigated when treating mice externally with the CDDO derivative RTA 408 (Nakagami and Masuda, 2016).

OSCC cells showed an increased sensitivity to CDDO-Me when compared to NHEK; IC_50_ values of CDDO-Me were roughly 3-fold lower in Cal-27 cells.

Furthermore, we postulate that the activation of the antioxidative Keap1/Nrf2 pathway, regularly attributed to CDDO-Me, may partially result from elevated ROS levels. Previous studies identified the direct interaction of synthetic triterpenoids with Keap1 to be responsible for the Nrf2 pathway initiation (Dinkova-Kostova et al., 2005). However, activated Nrf2 signaling was shown to be only partially involved in the up-regulation of HO-1 mediated by CDDO-derivatives (Liby et al., 2005). CDDO-Me is well known as a multifunctional drug, which initiates various energy-demanding processes like transcriptional pathways or apoptosis (Liby and Sporn, 2012). The required activation of mitochondrial metabolism for cellular energy supply accompanied by inevitable ROS generation may finally stimulate the up-regulation of antioxidative enzymes like HO-1.

However, further research is needed to illuminate the precise mechanisms and clinical relevance of CDDO-Me induced ROS generation in OSCC cells.

Recent scientific research highlighted the ambivalent role of ROS in cancer (Nogueira and Hay, 2013; Assi 2017). While elevated baseline ROS levels commonly found in highly metabolic active cancer cells are capable to mediate pro-oncogenic characteristics, excessive amounts of intracellular ROS were shown to induce senescence and/or apoptosis. The latter effect is widely exploited by conventional treatment regimens like chemotherapy or radiotherapy (Conklin 2004; Moreb et al., 2017). OSCC colony formation and proliferation was significantly reduced by combined treatment of IR with CDDO-Me pointing to radiosensitizing effects in OSCC cells. As these results were not confirmed or even contrarily in our primary cell line NHEK, we draw the conclusion that CDDO-Me could offer a radiosensitizing effect for certain OSCC cells while not impairing or even protecting the adjectant healty tissue. Finally, this could lead to lower required IR doses during radiotherapy of OSCC patients and thus to less side effects.

## Data Availability Statement

The raw data supporting the conclusions of this article will be made available by the authors on request, without undue reservation.

## Ethics Statement

CAM-experiments were performed in compliance with European and German laws for the protection of animals used for scientific purposes.

## Author Contributions

Following contributions to the present study were made by the respective scientist: HC, LS, and ES: conceptualization and methodology. HS: CAM experiments and statistics. HC, PT, HS, RA, DS, PM, and ES: data analysis; writing, reviewing, and editing of the manuscript. HC and ES: project administration and supervision.

## Funding

This study was funded by the German Ministry of Defense.

## Conflict of Interest

The authors declare that the research was conducted in the absence of any commercial or financial relationships that could be construed as a potential conflict of interest.

The handling editor DR declared a past co-authorship with one of the authors MP.

## Abbreviations

CAM, chorioallantoic membrane; CDDO-Me, 2-cyano-3,12-dioxooleana-1,9,-dien-28-oic acid methyl ester; DCFDA, dichlorodihydrofluorescein diacetate; eGFR, estimated glomerular filtration rate; IF, immunofluorescence; keap1, Kelch-like ECH-associated protein 1; min, minutes; NHEK, normal human epithelialkeratinocytes; NFκB, nuclear factor κB; Nrf2, nuclear factor erythroid 2–related factor; OSCC, oral squamous cell carcinoma; PE, plating efficiency; ROS, reactive oxygen species; RT, radiotherapy; SF, surviving fraction
